# RIN4 recruits the exocyst subunit EXO70B1 to the plasma membrane

**DOI:** 10.1093/jxb/erx007

**Published:** 2017-02-20

**Authors:** Peter Sabol, Ivan Kulich, Viktor Žárský

**Affiliations:** 1 Charles University in Prague, Viničná, Prague, Czech Republic; 2 Institute of Experimental Botany, Rozvojová, Prague, Czech Republic

**Keywords:** Autophagy, EXO70B1, EXO70B2, exocyst, plant immunity, RIN4, secretion

## Abstract

The exocyst is a conserved vesicle-tethering complex with principal roles in cell polarity and morphogenesis. Several studies point to its involvement in polarized secretion during microbial pathogen defense. In this context, we have found an interaction between the Arabidopsis EXO70B1 exocyst subunit, a protein which was previously associated with both the defense response and autophagy, and RPM1 INTERACTING PROTEIN 4 (RIN4), the best studied member of the NOI protein family and a known regulator of plant defense pathways. Interestingly, fragments of RIN4 mimicking the cleavage caused by the *Pseudomonas syringae* effector protease, AvrRpt2, fail to interact strongly with EXO70B1. We observed that transiently expressed RIN4, but not the plasma membrane (PM) protein aquaporin PIP2, recruits EXO70B1 to the PM. Unlike EXO70B1, RIN4 does not recruit the core exocyst subunit SEC6 to the PM under these conditions. Furthermore, the AvrRpt2 effector protease delivered by *P. syringae* is able to release both RIN4 and EXO70B1 to the cytoplasm. We present a model for how RIN4 might regulate the localization and putative function of EXO70B1 and speculate on the role the AvrRpt2 protease might have in the regulation of this defense response.

## Teaser

The exocyst subunit EXO70B1 is recruited to the plasma membrane by the immunity-related RIN4 protein and this change in localization is manipulated by a *Pseudomonas syringae* effector possibly to inhibit PAMP-triggered immunity.

## Introduction

The exocyst is a conserved protein complex involved in tethering secretory vesicles to the destination/target membrane. In plants, it is implicated in diverse cellular processes that lead to the asymmetric distribution of proteins and membranes within the cell (Elias *et al.*, 2003; Zárský *et al.*, 2009, 2013). Therefore, it is involved in processes of cell polarity establishment including root hair and pollen tube growth, polarized growth of stigmatic papillae, cytokinesis, localized deposition of seed coat pectin, transport of PIN auxin carriers to specific parts of the plasma membrane (PM), development of the periarbuscular membrane, maturation of the trichome cell wall, and probably polarized secretion of defense cargo during response to microbial pathogens (Synek *et al.*, 2006; Hála *et al.*, 2008; Kulich *et al.*, 2010, 2015; Pecenková *et al.*, 2011; Genre *et al.*, 2012; Drdová *et al.*, 2013; Zárský *et al.*, 2013; Zhang *et al.*, 2015). The molecular mechanism of exocyst action is believed to involve the tethering of secretory vesicles to the target membrane prior to SNARE-mediated fusion (Heider and Munson, 2012). Recently, a novel function for the exocyst subcomplex containing EXO70B1, SEC5, and EXO84 subunits has been assigned to autophagic membrane transport to the vacuole (Kulich *et al.*, 2013). Despite some progress, the previously described role for the EXO70E2 subunit in unconventional protein secretion and its relationship to the autophagic pathway remain controversial (Lin *et al.*, 2015). In yeast, EXO70 and SEC3 subunits are hypothesized to serve as landmarks for membrane targeting of the complex (Wu and Guo, 2015). Unlike yeast and mammals, plant genomes encode many isoforms of the EXO70 subunit (Elias *et al.*, 2003; Cvrčková *et al.*, 2012). They are differentially expressed during ontogenesis and in different tissues (Synek *et al.*, 2006; Li *et al.*, 2010), but several EXO70s are expressed in the same cell type, leading to the hypothesis that they might contribute to the definition of specific cortical PM domains within the same cell (Zárský *et al.*, 2009). It is also possible that some of them may have adopted functions different from vesicle tethering during exocytosis. According to the publicly available data, several of the isoforms are transcriptionally up-regulated by pathogens or pathogen elicitors (Hruz *et al.*, 2008), and there is further evidence that points to a substantial contribution of the exocyst to plant defense against microbial pathogens (Pecenková *et al.*, 2011; Stegmann *et al.*, 2012). This engagement in the competition with parasites is also the best explanation for the extraordinary dynamics of EXO70 family evolution (Pecenková *et al.*, 2011; Cvrčková *et al.*, 2012).

It is therefore not surprising that a growing number of studies indicate a direct involvement of the exocyst complex in polarized secretion and signaling during the defense against microbial pathogens. While the *exo70H1* Arabidopsis mutant is more susceptible to infection by the bacterial pathogen *Pseudomonas syringae* pv *maculicola*, the *exo70B2* mutant is more susceptible to the virulent *Pseudomonas syringae* DC3000 (Pto DC3000), *P. syringae* pv *maculicola*, and the oomycete infection caused by *Hyaloperonospora arabidopsidis* (Pecenková *et al.*, 2011; Stegmann *et al.*, 2012). In addition, *exo70B2* forms abnormal papillae during the infection caused by the fungal pathogen *Blumeria graminis* f. sp. *hordei*. The closest homolog of EXO70B2 is the EXO70B1 exocyst subunit, which, besides its role in autophagy, is also engaged in defense against phytopathogens. The Arabidopsis *exo70B1* mutant was reported to be less resistant to Pto DC3000 than the wild type (Stegmann *et al.*, 2013), while it was found to be more resistant to the same pathogen and the Pto DC3000 bacterium expressing the AvrRpt2 effector (Zhao *et al.*, 2015). Differences between the two studies were thought to be due to the different growth conditions used; however, the same mutant displays enhanced resistance to the oomycete *H. arabidopsidis* and the adapted powdery mildew *Golovinomyces cichoracearum*, apparently due to the lower threshold for hypersensitive response activation (Stegmann *et al.*, 2013; Zhao *et al.*, 2015). One member of the cereal-specific EXO70 subfamily is involved in defense against *B. graminis* (Ostertag *et al.*, 2013), and also a core subunit was reported to be targeted by pathogen effectors—the SEC5 exocyst subunit of *Nicotiana benthamiana* interacts with the *Phytophtora infestans* AVR1 effector, resulting in increased sensitivity of the host plant towards the pathogen (Du *et al.*, 2015).

While looking for Arabidopsis exocyst subunit interactors using a yeast two-hybrid (Y2H) screen, we found a binding partner for some of the EXO70 subunits which belongs to the NOI protein family. Based on this observation, we also tested the RPM1 INTERACTING PROTEIN 4 (RIN4) protein for possible exocyst subunit interactions and found that it interacted with the EXO70B1 exocyst subunit. RIN4 is a member of a larger NOI protein family that shares either one or both conserved NOI (nitrate-induced) domains with a conserved cleavage site [termed the RIN4 cleavage site (RCS)] for the bacterial protease AvrRpt2 (Afzal *et al.*, 2013). Besides AvrRpt2, RIN4 is a target of at least three other effector proteins in Arabidopsis that induce either phosphorylation or ADP-ribosylation, and thus, presumably, inhibit the pathogen-associated molecular pattern (PAMP)-triggered immunity (PTI) response. In the presence of the corresponding resistance (R) proteins, however, effector-triggered immunity (ETI) is initiated, which often culminates in a hypersensitive cell death response. In the case of AvrRpt2, cleavage of RIN4 at RCSs activates the RPS2 R protein (Mackey *et al.*, 2002; Wilton *et al.*, 2010; Afzal *et al.*, 2011). RIN4 homologs in soybean form a heteromeric complex in which only some components interact with the soybean RPS2 ortholog (Selote and Kachroo, 2010). Recently, a new study showed that the AvrRpt2 protease is also able to inhibit PTI independently of RIN4 (Eschen-Lippold *et al.*, 2016).

Although it is speculated that the role of the putative NOI–exocyst interaction is important for pathogen responses in plants (Da Cunha, 2009; Afzal *et al.*, 2013), the actual mechanism through which RIN4 and possibly other NOI proteins influence exocyst function during a defense response is unknown. Because both RIN4 and the exocyst operate at the PM, we investigated the role RIN4 has in the regulation of exocyst localization or function. Moreover, due to the autophagic origin of some of the cellular compartments involved in polarized secretion during the defense response, we speculate that the autophagic and putative defense secretion roles of EXO70B1 might be connected.

## Materials and methods

### Co-immunoprecipitation

For co-immunoprecipitation (co-IP) experiments, we used 2–3 leaves of *N. benthamiana* transiently expressing red fluorescent protein (RFP)–EXO70B1, green fluorescent protein (GFP)–RIN4, and free yellow fluorescent protein (YFP) under the control of the ubiquitin promoter (in pUB vectors; Grefen *et al.*, 2010), RFP–EXO70B2 under the control of the 35S promoter (pH7WGR2 vector; Pecenková *et al.*, 2011), and from leaves from plants infiltrated with P19 vector only as a negative control. For protein extraction, we ground 3–4 agroinfiltrated leaves in liquid nitrogen and transferred the resulting powder into the ice-cold extraction buffer with added protease inhibitors (Roche). We vortexed the lysates briefly and centrifuged them at 3700 *g*, 4 °C for 15 min, and subsequently centrifuged the supernatant for another 10 min at 10 000 *g*, 4 °C. We then used a µMACS GFP Isolation Kit (Miltenyi) for co-IP. We used a modified extraction buffer based on the Miltenyi kit Lysis Buffer that contained only 0.1% Triton X-100 (compared with 1% in kit buffer), as we experienced non-specific binding of proteins to the magnetic beads at high Triton X-100 concentration as previously described (Avila *et al.*, 2015). Eluates were resolved on a 10% polyacrylamide gel and blotted onto a nitrocellulose membrane. Proteins were then detected on the membrane using anti-RFP (ChromoTek), anti-hemagglutinin (HA; Cell Signaling), and horseradish peroxidase (HRP)-conjugated anti-GFP (Miltenyi) antibodies.

### Transient expression of proteins in *Nicotiana benthamiana*

Transient expression was performed as previously described (Pecenková *et al.*, 2011) with slight modification to the composition of the infiltration buffer (50 mM MES pH 5.6, 2 mM Na_3_PO_4_, 0.5% glucose, and 100 μM acetosyringone). For co-IP, we extracted proteins from leaves 3 d after infiltration. For microscopic observations, we used GFP–RIN4 under the control of the ubiquitin promoter, RFP–EXO70B1 (coding sequence) under the control of the ubiquitin promoter, and RFP–EXO70B2 under the control of the 35S promoter (pH7WGR2 vector; used in Pecenková *et al.*, 2011). We cloned the GFP–RIN4 construct from a cDNA clone in the pUNI51 vector obtained from the Arabidopsis Biological Resource Center, into the pENTR1A vector and then into the pUBN-GFP destination vector (Grefen *et al.*, 2010). We also cloned the EXO70B1 coding sequence into the pENTR1A vector using primers in Table 1, and then into the pUBN-RFP destination vector (Grefen *et al.*, 2010). The fluorescent constructs were observed in plants 2–3 d after infiltration. For co-localization analysis, we employed a 35S:PIP2;1–GFP construct (in the pGWB5 vector) as a PM-localized protein (Boursiac *et al.*, 2005). For further recruitment studies, we cloned EXO70B1 with a C-terminal cyan fluorescent protein (CFP) under the 35S promoter into the pB7m34GW MultiSite Gateway destination vector. The 35S:SEC6–RFP construct was kindly provided by Tamara Pečenková, and the GFP–SYP121 construct by Mike Blatt’s laboratory.

**Table 1.** List of primers

RFP-EXO70B1	B1 CDS ECOrevstop	AAAGTCGACATGGCGGAGAATGGTGAAG
	B1cdsSAL1	TTTGAATTCCTTCATTTTCTTCCCGTGGTAGTC
RCS2_C in pGADT7 (without the C-terminus) with the C-terminus	RIN4_RCS2_C_T7_for	TGCGGATCCTCATGGACTGGGACGAGAACA
	RIN4_RCS2_C_T7_rev	CAAGTCGACTTCATGAGGAAGTGTTGTTCG
	RIN4 FR pGAD for	AGGGATCCCCATGGACTGGGAC
RIN4 CDS in pGADT7	RIN4 full pGAD for	AGGGATCCCCATGGCACGTTC
	RIN4 full pGAD rev	CAGTCGACCTCATTTTCCTCCAAAG
RIN4 N-RCS2 in pENTR1A	RIN4_N_RCS2_SalI	TTCGTCGACCATCAAACCGAATTTAGGCACCACT
	RIN4_N_RCS2_BamHI	GAGGGATCCCCATGGCACGTTCGAATGTAC
EXO70B1 coding sequence with promoter	B1 for	CGGGAATTCAGAGGATAGGAATATATAAAT
	B1 rev	ACCGTCGACTTTCTTCCCGTGG
mRuby2 cloning into TagRFP-AS-N	mRUBY2 AGEI for	ACCGGTAATGGTGTCTAAGGGCGAAGAG
	mRUBY2 NOT1 rev	TTTGCGGCCGCTTACTTGTACAGCTCGTCCATCC

### Cloning of EXO70B1–mRuby2 with the EXO70B1 promoter

The EXO70B1 coding sequence together with the EXO70B1 promoter was cloned into the Gateway® TagRFP-AS-N vector (Evrogen) using the primers in Table 1. The TagRFP sequence was then replaced by mRuby2 from the cDNA3.1-Clover-mRuby2 vector using the primers in Table 1. Vector pcDNA3.1-Clover-mRuby2 was a gift from Kurt Beam (Addgene plasmid # 49089). The LR reaction was performed subsequently between this vector and a modified pBGW vector (Karimi *et al.*, 2002) containing the transcription terminator. To clone the transcription terminator into the pBGW vector, a *Pst*I fragment (with attr2 and a terminator) of the pUB destination vector (Grefen *et al.*, 2010) was cloned into the original pBGW vector.

### Yeast two-hybrid assay

NOI6 and different RIN4 constructs were cloned into the pGADT7 vector using the primers in Table 1. All the other exocyst constructs and the Y2H assay have been described previously (Hála *et al.*, 2008). Briefly, yeast transformed with the respective constructs were plated first on plates without leucine and tryptophan and then in a 10× dilution series starting with OD_600nm_=0.1 on plates without leucine, tryptophan, histidine, and adenine to test for protein–protein interactions.

### Confocal microscopy analysis

For image acquisition, we used a Leica TCS SP2 confocal microscope with a ×63/1.2 water immersion objective, a Zeiss LSM 880 confocal microscope with a ×63/1.2 water immersion objective, and a Nikon TE200e with a Yokogawa Andor spinning disc unit. The GFP constructs were excited at 488 nm and detected at 505–530 nm, and RFP constructs were excited at 560 nm and detected at 600–620 nm.

### Quantification of fluorescence signals

To calculate membrane to cytoplasm fluorescent signal ratios, we measured the fluorescence intensity in *N. benthamiana* cells transiently expressing the respective fluorescent constructs. For this, we constructed *Z*-stacks of cells from which we further chose projections only from the medial plane of the cell to avoid mistaking the PM surface for a cytoplasmic signal. Using the Fiji software (Schindelin *et al.*, 2012), we first measured the raw integrated density divided by the region of interest (ROI) area, and then calculated the average value of this ratio from five different regions within a cell. We determined the membrane to cytoplasm ratio as a mean value from 11 cells for each combination of constructs.

### Pseudomonas syringae assay

We first infiltrated tobacco leaves with *Agrobacterium* strains harboring the different constructs described above. One day after agroinfiltration, we grew an overnight liquid culture of Pto DC3000 strains HrpH– (a mutant for the type III secretion system) and AvrRpt2 in LB medium with appropriate antibiotics. Cultures were pelleted and washed with 10 mM MgCl_2_ to remove the antibiotics, and then diluted to the final OD_600 nm_=0.1 in 10 mM MgCl_2_. We infiltrated this solution into already agroinfiltrated leaves and observed the fluorescence 18–20 h later. No obvious cell death symptoms were macroscopically visible in such treated leaves.

### DTT treatment

For the evaluation of the effect of DTT on protein localization, we infiltrated a 50 mM aqueous DTT solution into *Agrobacterium*-infiltrated *N. benthamiana* leaves 3 d after agroinfiltration and observed the fluorescence signal 3 h after DTT infiltration.

## Results

### Exocyst subunit EXO70B1 interacts with RIN4, but not with RIN4 fragments

**Fig. 1.**
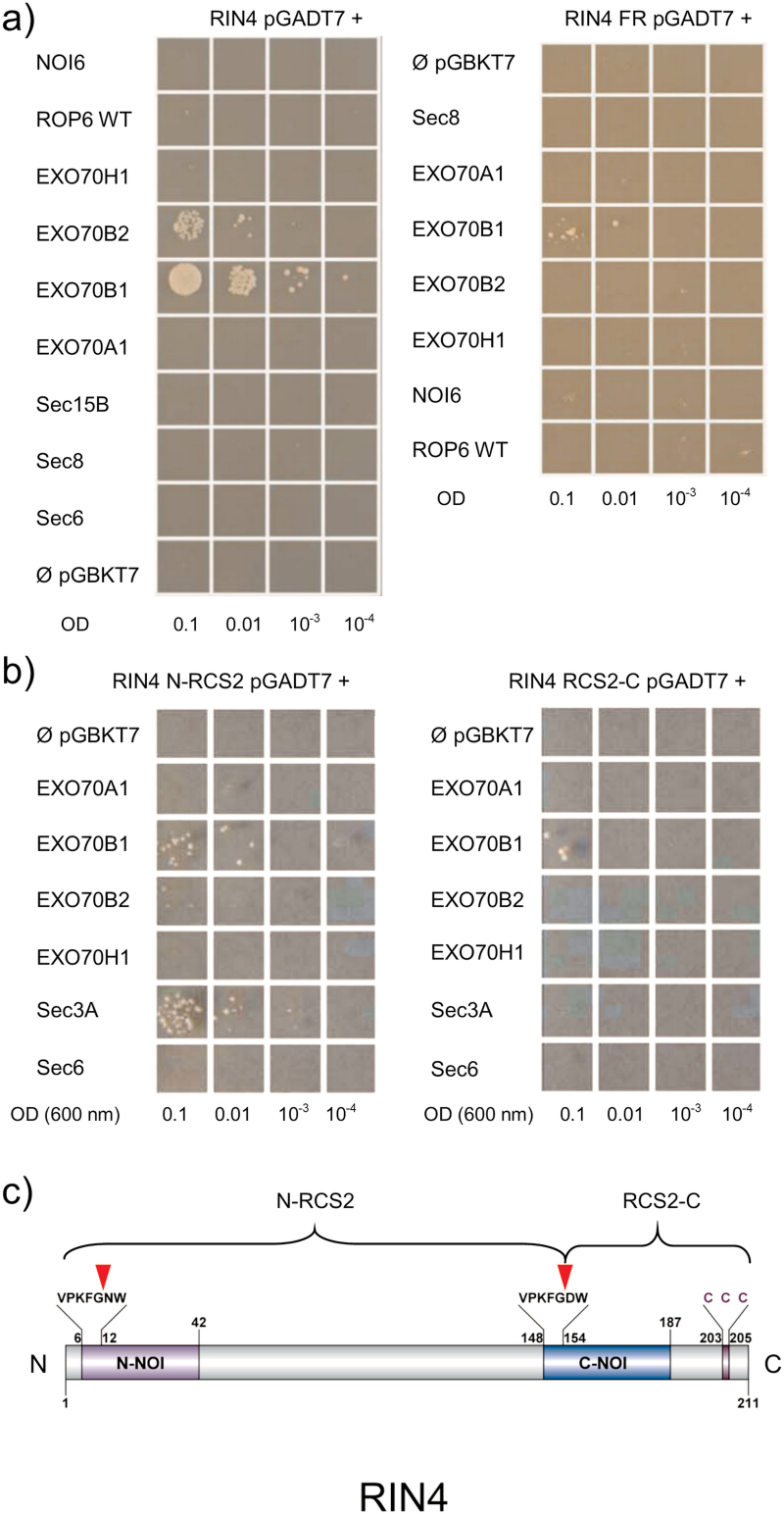
Yeast-two hybrid assays showing interactions of exocyst subunits with RIN4. (a) Full-length RIN4 protein (on the left) and the C-terminal membrane-anchored RIN4 fragment (on the right). (b) An N-terminal fragment and a C-terminal fragment without palmitoylation sites. SEC3A yeasts autoactivate expression of selection markers (as shown by Hála *et al.*, 2008). (c) The overall structure of RIN4. The AvrRpt2 cleavage consensus sequences (RCS) within NOI domains are shown above the bar scheme, with actual cleavage sites indicated by triangles. C-terminal cysteine residues responsible for membrane anchoring are shown as CCC above the bar scheme. The range of N- (N-RCS2) and C- (RCS2-C) terminal RIN4 fragments used in Y2H experiments is also depicted above the bar scheme of the protein. The model of RIN4 was drawn with CSS-Palm software (Ren *et al.*, 2008). (This figure is available in colour at *JXB* online.)

In our Y2H screen, we identified the NOI6 protein as an interactor of the EXO70A1 exocyst subunit (see Supplementary Fig. S1 at *JXB* online). NOI6 (At5g64850) is a member of a larger family of NOI proteins in Arabidopsis (for a recent review, see Afzal *et al.*, 2013). This led us to test whether any of the subunits of the Arabidopsis exocyst complex would interact with the RIN4 protein, which is the most studied member of the NOI family. We therefore cloned the RIN4 coding sequence into a Y2H prey vector, several of the exocyst subunits into bait vectors, and subsequently tested for pairwise interactions. Interestingly, we observed an interaction between the full-length RIN4 protein and EXO70B1. A weak interaction was also observed with the EXO70B2 subunit (Fig. 1a). These are two closely related paralogs within the B clade of the Arabidopsis EXO70 family that share some sequence similarity at the protein level (for a recent review, see Cvrčková *et al.*, 2012). None of the other EXO70 paralogs tested showed this interaction with RIN4.

When the bacterial pathogen *P. syringae* injects its AvrRpt2 effector protein into the plant cell, it cleaves RIN4 at two well-conserved cleavage sites, which produces three fragments; two fragments are subsequently released into the cytoplasm and suppress PTI (Afzal *et al.*, 2011). We therefore tested the interaction with a C-terminal RIN4 fragment that mimics cleavage by the AvrRpt2 protease at the C–NOI cleavage site (Kim *et al.*, 2005; Afzal *et al.*, 2011). Interactions with this cleavage product were lost and only EXO70B1 showed a very weak interaction. Similarly, the N-terminal RIN4 fragment that mimics cleavage at the N–NOI cleavage site also interacts weakly with only EXO70B1, although somewhat more strongly than the C-terminal fragment (Fig. 1b).

**Fig. 2.**
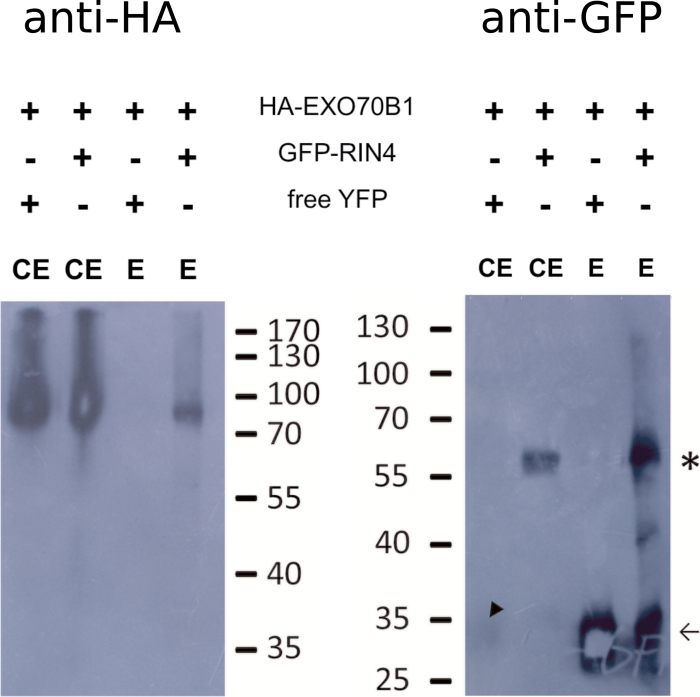
Co-immunoprecipitation assay (co-IP) showing an interaction between HA-EXO70B1 and GFP–RIN4 proteins transiently expressed in *N. benthamiana*. Proteins were immunoprecipitated with anti-GFP magnetic beads, and EXO70B1 was detected with anti-HA antibody. Experiments were performed three times with similar results. Marker sizes are shown in kDa next to the blots. Left: detection of HA-EXO70B1 in total cell extracts (CE) from plants co-expressing free YFP; in total cell extracts from plants co-expressing GFP–RIN4, in eluate (E) after co-IP with free YFP, and in eluate after co-IP with GFP–RIN4. Right: detection of GFP/YFP in plants expressing HA-EXO70B1 and co-expressing YFP in total cell extract (CE), GFP–RIN4 in total cell extract, YFP in eluate (E) after co-IP, and GFP–RIN4 in eluate after co-IP. The arrow marks bands corresponding to free YFP, and the asterisk marks the position of GFP–RIN4. (This figure is available in colour at *JXB* online.)

We confirmed EXO70B1 Y2H interactions with the full-length RIN4 using a co-IP assay with transiently expressed proteins in *N. benthamiana* leaves. With anti-GFP magnetic beads, we were able to precipitate HA-tagged EXO70B1 protein together with GFP–RIN4, but not with the free YFP control (Fig. 2). Together, our data indicate an interaction between EXO70B1 and RIN4 proteins.

### RIN4 recruits EXO70B1 to the PM

**Fig. 3.**
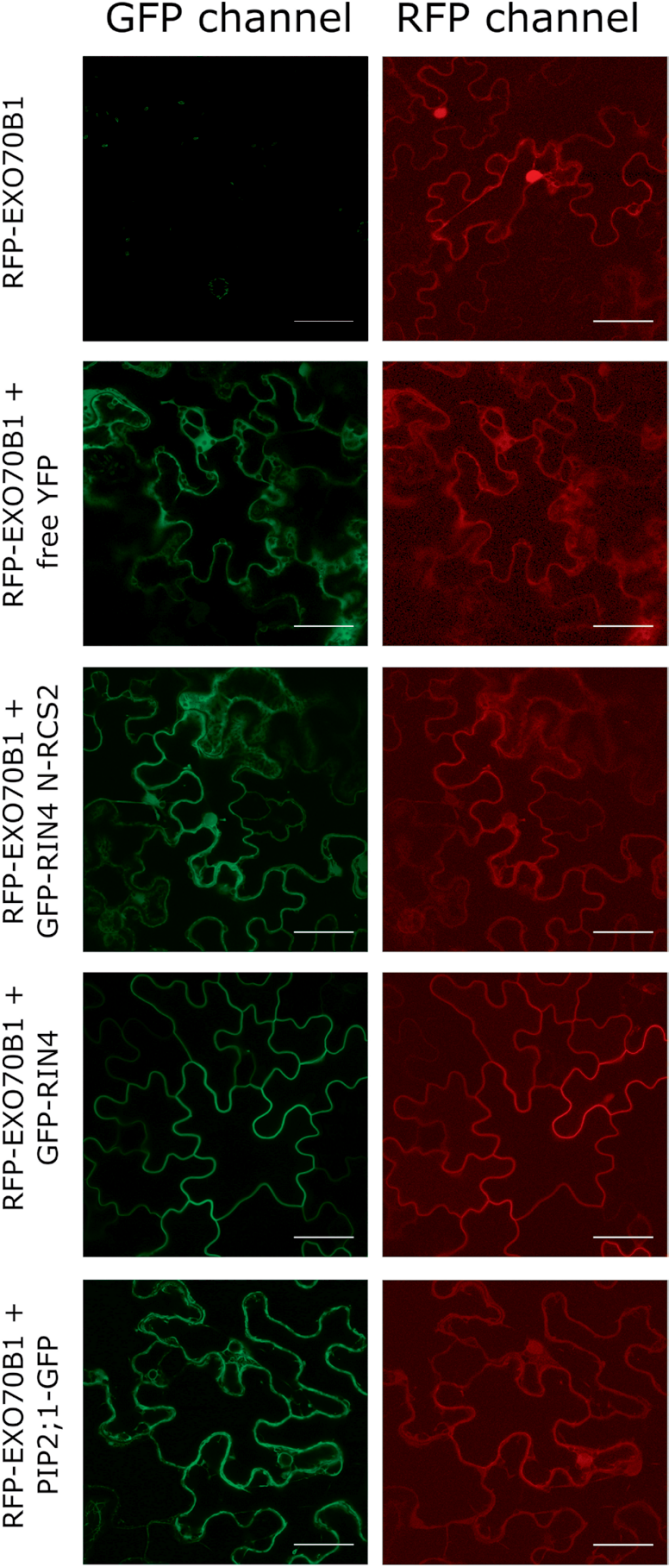
RIN4 recruits EXO70B1 to the plasma membrane. Shown are the confocal microscopy images of fluorescently labeled constructs transiently expressed in *N. benthamiana* leaves. While RFP–EXO70B1 alone (upper two panels) localized mainly to the cytoplasm and nucleus, when co-expressed with GFP–RIN4, RFP–EXO70B1 was almost exclusively localized to the plasma membrane. In contrast to RIN4, aquaporin PIP2;1–GFP did not change the subcellular localization of RFP–EXO70B1. RFP–EXO70B1 without RIN4 co-localized in the cytoplasm with both free YFP and the N-terminal RIN4 fragment (N-RCS2). Scale bars=50 µm.

As the RIN4 protein is localized to the PM and one of the putative functions of EXO70B1 would be the targeting of vesicles to the pathogen attack site based on its immunity-related phenotypes (Stegmann *et al.*, 2012; Zhao *et al.*, 2015), we hypothesized that RIN4 might be required for the EXO70B1 protein and possibly also the whole exocyst complex function at the PM. Due to the presence of several NOI proteins in the Arabidopsis proteome with potential functional redundancy and the difficulty of working with multiple gene knockouts, we used the *N. benthamiana* heterologous model system for transient expression and cellular dynamic studies. When we expressed RFP–EXO70B1 under the ubiquitin promoter in *N. benthamiana* leaves, we observed a strong fluorescence signal in the cytoplasm, nucleus, and at the PM, essentially in accordance with the previously published localization patterns (Hong *et al.*, 2016; Fig. 3). We confirmed the cytoplasmic localization of RFP–EXO70B1 by co-expression with both free YFP and the previously published cytoplasmic N-terminal GFP–RIN4 fragment, which is normally released to the cytoplasm after AvrRpt2 cleavage at the RCS2 cleavage site (Takemoto and Jones, 2005; Afzal *et al.*, 2011).

Interestingly, when we co-expressed RFP–EXO70B1 with GFP–RIN4 under the ubiquitin promoter, EXO70B1 localized almost exclusively to the PM (Fig. 3). This localization was dependent on the strength of GFP–RIN4 expression, with stronger expression resulting in more RIN4 protein on the PM, and subsequently more EXO70B1 on the PM. Quantification of membrane to cytoplasm fluorescence ratios revealed a significant difference between EXO70B1 PM localization when EXO70B1 was expressed alone as compared with when EXO70B1 was co-expressed with RIN4 (Fig. 6a). We further wanted to know whether the effect of EXO70B1 recruitment to the PM was specific for the RIN4 protein. We therefore expressed another PM-localized protein, PIP2;1 aquaporin, and checked whether it influenced EXO70B1 localization. When PIP2;1–GFP was expressed transiently in *N. benthamiana* under the 35S promoter, it localized not only to the PM, but also to the cytoplasm and a structure possibly representing the endoplasmic reticulum. Nevertheless, when we co-expressed RFP–EXO70B1 together with PIP2;1–GFP, EXO70B1 localization was still cytoplasmic (Fig. 3), confirming that RIN4 specifically recruits EXO70B1 to the PM. Similarly, EXO70B1 partially co-localized with GFP–SYP121 expressed under the ubiquitin promoter in the PM. However, GFP–SYP121 did not recruit EXO70B1 to the PM (Supplementary Fig. S2).

**Fig. 4.**
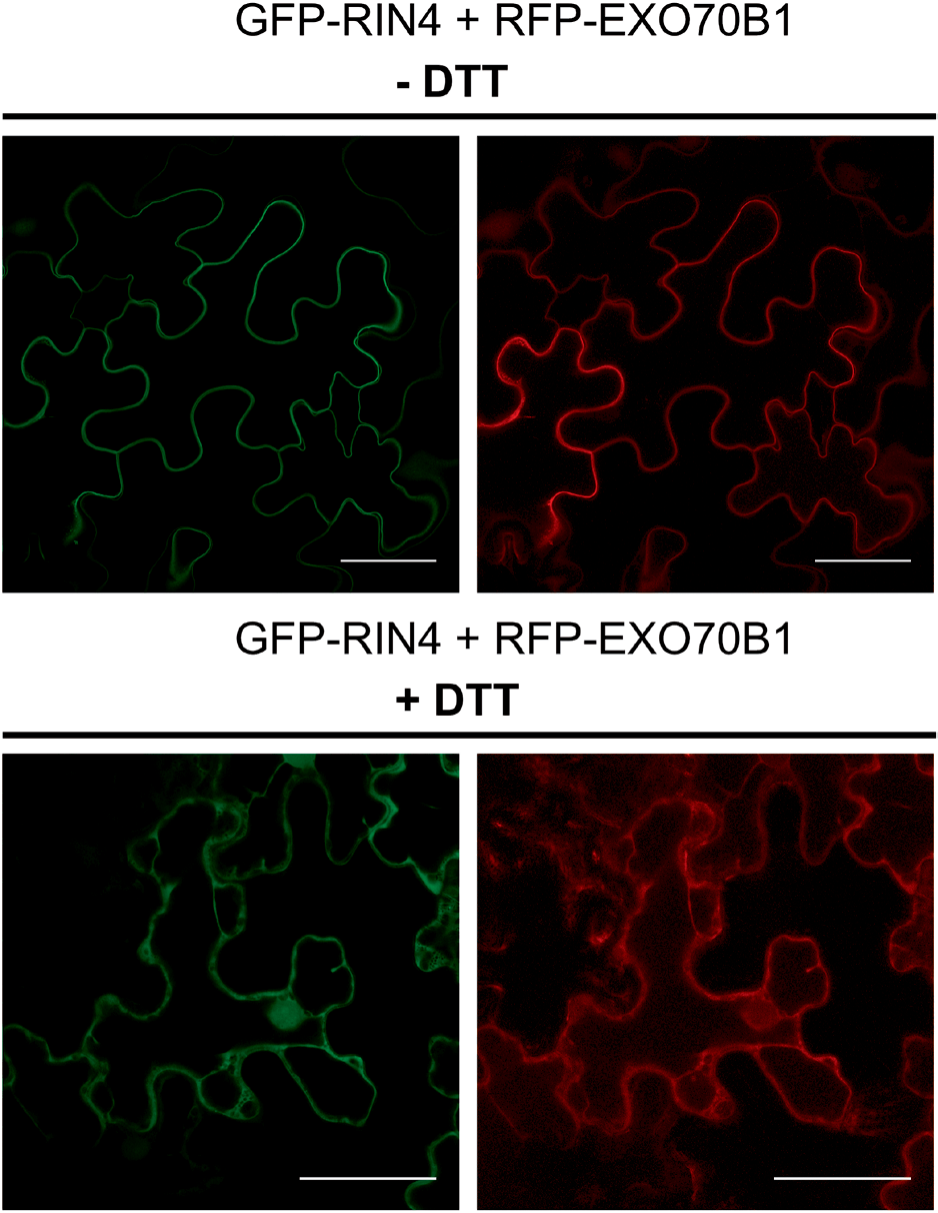
DTT causes relocalization of both GFP–RIN4 and RFP–EXO70B1 to the cytoplasm. Shown are the confocal microscopy images of fluorescently labeled constructs transiently expressed in *N. benthamiana* leaves. Upper panels depict control cells; lower panels show cells treated with 50 mM DTT. Scale bars=50 µm.

Application of DTT for 3 h by infiltration into *N. benthamiana* leaves caused relocalization of GFP–RIN4 to the cytoplasm, possibly due to interference with palmitoylation of C-terminal cysteinyl residues (Fig. 4). Although we cannot rule out a general effect of DTT on disulfide bond formation and, thus, the tertiary structure of the protein, disulfide bond prediction software did not predict any disulfide bonds in RIN4 or EXO70B1 proteins, while the other model predicted some disulfide bonds in EXO70B1 only (Ceroni *et al.*, 2006; Ferrè and Clote, 2006). Nevertheless, RFP–EXO70B1 also translocated from the membrane to the cytoplasm under these conditions, as compared with the untreated control cells. These results suggest that RIN4 is mainly responsible for membrane localization of EXO70B1.

### Pseudomonas AvrRpt2 releases both RIN4 and EXO70B1 from the PM to the cytoplasm

**Fig. 5.**
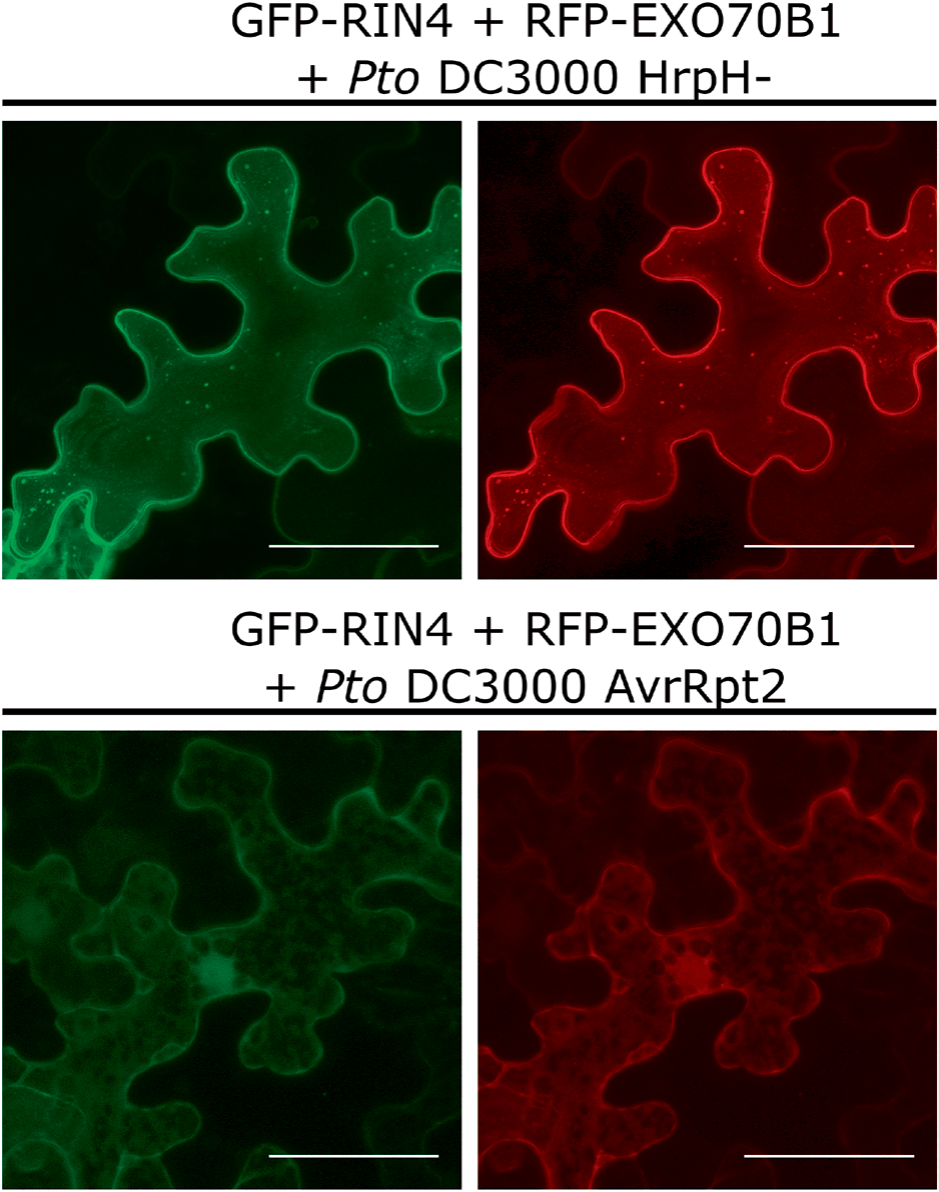
*P. syringae* AvrRpt2 causes relocalization of both GFP–RIN4 and RFP–EXO70B1 to the cytoplasm. Shown are *Z* projections of confocal images of cells transiently expressing GFP–RIN4 (left panels) together with RFP–EXO70B1 (right panels) that were co-infiltrated either with the mutant strain of *Pseudomonas* (*Pto* DC3000 HrpH–; upper two panels) or with a strain harboring the AvrRpt2 protease (*Pto* DC3000 AvrRpt2; lower two panels). See the Materials and Methods for further details. Scale bars=50 µm.

Based on our interaction and localization data, we reasoned that the delivery of the AvrRpt2 protease, which cleaves RIN4 into fragments (Afzal *et al.*, 2011), could also release EXO70B1 from the PM. Indeed, we observed RFP–EXO70B1 signal mostly in the cytoplasm in most of the fluorescent agroinfiltrated *N. benthamiana* leaf cells after we co-infiltrated a *P. syrinage* strain expressing the AvrRpt2 protease. Both GFP–RIN4 and RFP–EXO70B1 were released to the cytoplasm in cells where AvrRpt2 had been delivered. In contrast, both GFP–RIN4 and RFP–EXO70B1 stayed at the PM when we co-infiltrated the *P. syringae* mutant strain HrpH– that is deficient in the type III secretion system (Fig. 5). These results support the notion that PM localization of EXO70B1 is to a large extent dependent on intact RIN4 protein.

### RIN4 does not recruit EXO70B2 to the PM

**Fig. 6.**
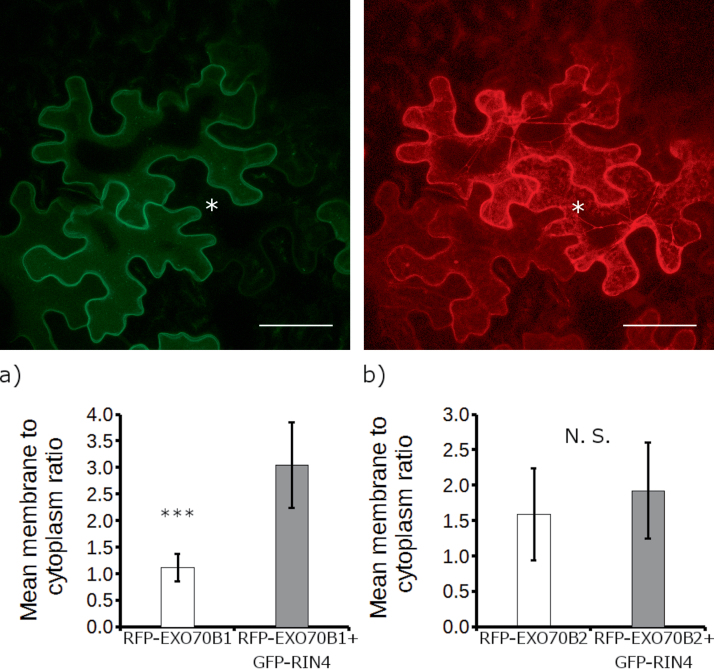
RIN4 does not recruit EXO70B2 to the plasma membrane. GFP–RIN4 (upper left panel) was overexpressed together with RFP–EXO70B2 (upper right panel) in *N. benthamiana* leaves. Asterisks mark a cell expressing RFP–EXO70B2 only. Panels are *Z* projections of 17 confocal sections. Scale bar=50 µm. Quantification of the membrane to cytoplasm fluorescence ratio from constructs transiently expressed in *N. benthamiana* leaves is shown in the bottom part of the figure. Fluorescence was measured as described in the Materials and Methods in the cytoplasmic strands and the membrane portion of the cells. Shown are the average and the SD (error bars) from 11 cells for each combination. Cytoplasmic strands were seen only occasionally in (a) with most RFP–EXO70B1 signal being on the plasma membrane when co-expressed with GFP–RIN4. The average ratio for this combination is therefore underestimated. Three asterisks denote a significant difference as determined by a *t*-test (*P*-value <0.001) and N.S. indicates a difference that is not statistically significant (*t*-test *P*-value >0.05).

The closest homolog of EXO70B1 in Arabidopsis is the EXO70B2 exocyst subunit, which also showed some interaction with RIN4 in our Y2H assay (Fig. 1). We therefore asked if EXO70B2 is also recruited by RIN4 to the PM. When transiently expressed under the 35S promoter in *N. benthamiana* leaves, EXO70B2 localized mostly to the cytoplasm, as previously reported (Pecenková *et al.*, 2011), with some weak PM signal. Unlike EXO70B1, RFP–EXO70B2 stayed in the cytoplasm even when it was co-expressed with GFP–RIN4 (Fig. 6). Additionally, fluorescence signal quantification did not show any significant difference between EXO70B2 expressed alone and EXO70B2 co-expressed with RIN4 (Fig. 6b). Therefore, EXO70B2 is not recruited to the PM by RIN4.

### SEC6 core exocyst subunit is also not recruited to the PM by RIN4

**Fig. 7.**
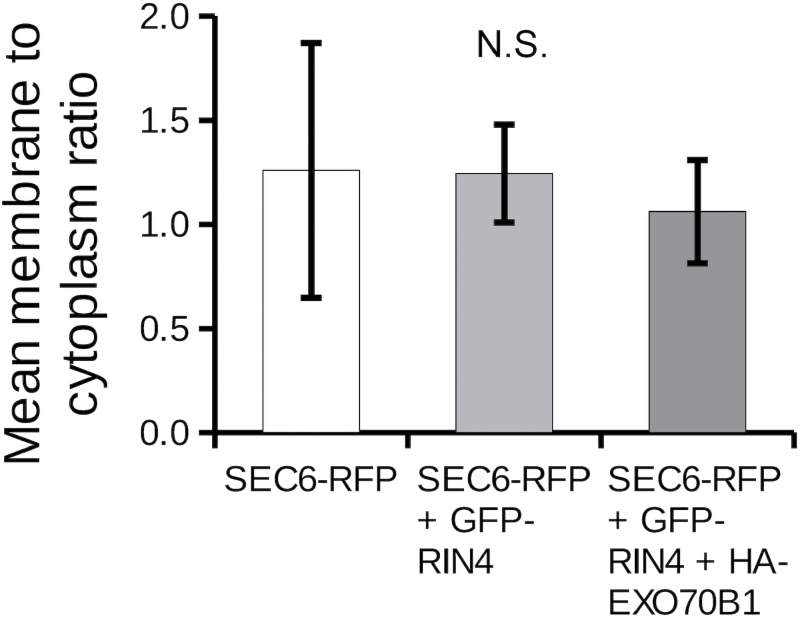
Neither GFP–RIN4 nor GFP–RIN4 co-expressed with HA-EXO70B1 recruit SEC6–RFP to the PM. Shown is the quantification of membrane to cytoplasm fluorescence ratio of constructs transiently expressed in *N. benthamiana* leaves. Fluorescence was measured as described in the Materials and Methods in the cytoplasmic strands and the membrane portion of the cell. Depicted are the means and the SD (error bars) from eight (SEC6–RFP alone and with GFP–RIN4) and nine (SEC6–RFP with GFP–RIN4 and HA-EXO70B1) cells. Differences between the means are not statistically significant (N.S.; ANOVA *P*-value=0.599).

**Fig. 8.**
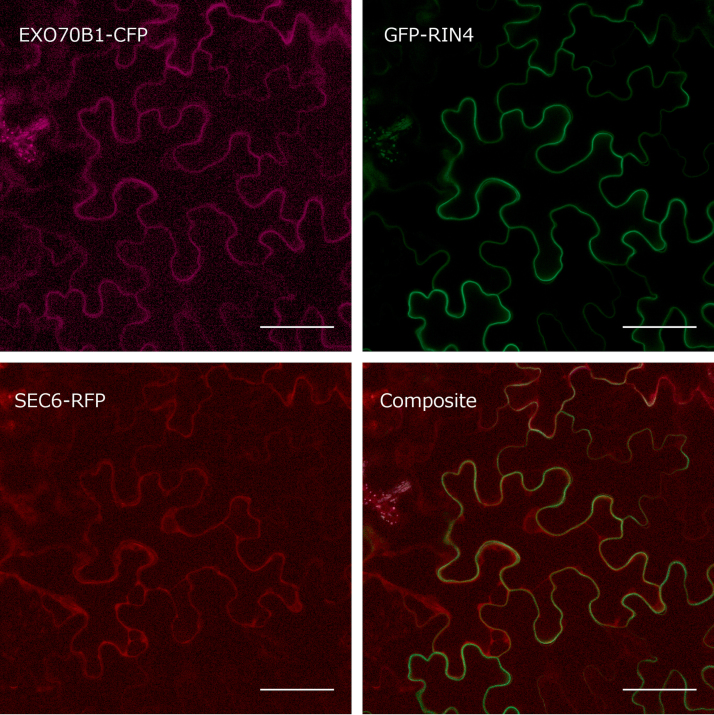
RIN4 does not recruit SEC6 even in the presence of EXO70B1. EXO70B1–CFP was co-expressed with GFP–RIN4 and SEC6–RFP transiently in *N. benthamiana* leaves. Shown are the confocal images and composite images. Cytoplasmic strands are clearly visible in the SEC6–RFP channel. Scale bar=50 µm.

To determine whether RIN4 recruits EXO70B1 to the PM as a part of the exocyst holocomplex, we investigated SEC6 core exocyst subunit subcellular localization. SEC6–RFP (Fendrych *et al.*, 2013) was expressed transiently under the 35S promoter in *N. benthamiana* leaves and localized almost exclusively to the cytoplasm with minimal PM signal. When co-expressed with GFP–RIN4, SEC6–RFP remained in the cytoplasm. We previously observed that SEC6 interacts with EXO70B1 (Kulich *et al.*, 2013) and therefore wondered if SEC6 PM localization would require co-expression of the *Arabidopsis thaliana* EXO70B1. However, when we co-expressed SEC6–RFP together with GFP–RIN4 and HA-tagged EXO70B1, SEC6–RFP still showed a mostly cytoplasmic signal (Fig. 7). Along with HA-EXO70B1, we also cloned and transiently expressed EXO70B1 with a C-terminal CFP together with GFP–RIN4 and SEC6–RFP; however, in this combination, GFP–RIN4 was unable to recruit SEC6–RFP to the PM (Fig. 8). Thus, neither GFP–RIN4 alone nor combined with HA-EXO70B1 or EXO70B1–CFP is sufficient to recruit SEC6–RFP to the PM.

### In Arabidopsis, RIN4 recruits EXO70B1 to the PM in guard cells

**Fig. 9.**
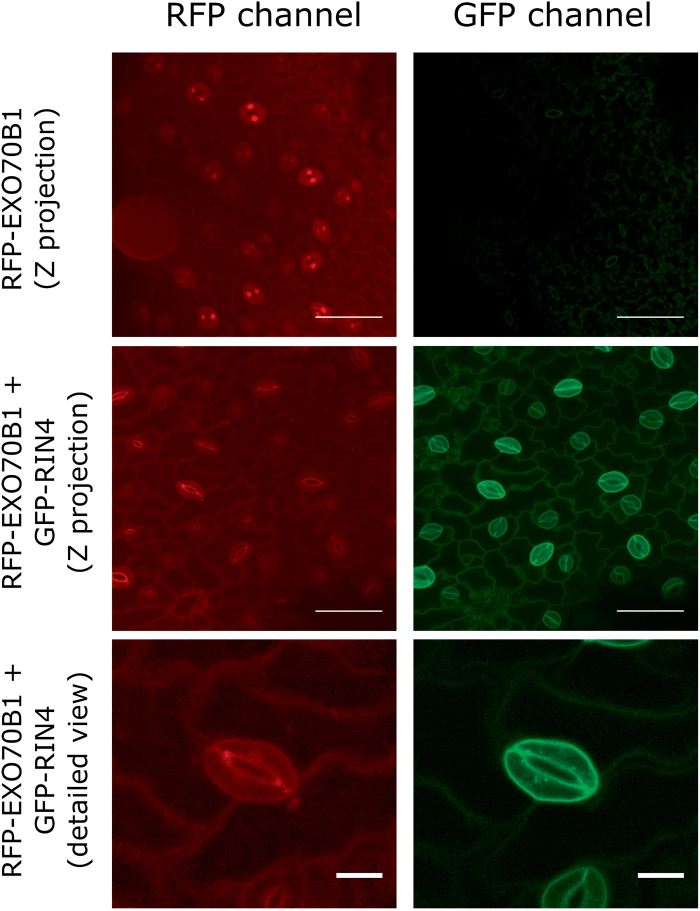
RIN4 recruits EXO70B1 to the PM in Arabidopsis stomatal guard cells. Confocal images of epidermal pavement and guard cells of the first true leaves of Arabidopsis seedlings show red fluorescence of RFP–EXO70B1 and green fluorescence of GFP–RIN4. Note the cytoplasmic and nuclear signal when RFP–EXO70B1 is expressed alone. Images in the upper two rows are maximal projections of 24 (RFP–EXO70B1) and nine (RFP–EXO70B1+GFP–RIN4) optical sections, respectively. The scale bar is 50 µm for the images in the upper two rows and 10 µm for the third row.

Finally, we were interested in determining how the cellular localization of the RFP-tagged EXO70B1 exocyst subunit is influenced by GFP–RIN4 expression in Arabidopsis. In the wild-type (WT) background, RIN4, and possibly other NOI proteins that might interact with EXO70B1 and influence its localization, is endogenously expressed. We therefore did not expect a large change in EXO70B1 localization in WT Arabidopsis after GFP–RIN4 overexpression. This turned out to be the case for epidermal pavement cells of the first true leaves in 7-day-old Arabidopsis seedlings, where RFP–EXO70B1 expressed under the ubiquitin promoter localized mostly to the PM (Fig. 9). This localization remained unchanged even in plants co-expressing GFP–RIN4 under the ubiquitin promoter. Surprisingly, this pattern was quite distinct from RFP–EXO70B1 localization in stomatal guard cells, where it localized mostly to the cytoplasm and the nucleus, similar to the localization in the *N. benthamiana* transient system, where EXO70B1 was recruited to the PM in plants overexpressing GFP–RIN4 (Fig. 9).

To see the effect of *rin4* mutation on EXO70B1 PM localization, we cloned the EXO70B1–mRuby2 construct under its own promoter and compared its localization in the WT and *rin4 rps2* mutant. Consistent with the redundancy in the NOI protein family, we did not observe any difference in localization between the WT and *rin4 rps2* mutant cotyledon cells of 7-day-old seedlings. EXO70B1–mRuby2 localized to the PM and the cytoplasm in both genotypes (Supplementary Fig. S3). In comparison with the ubiquitin promoter-driven construct, however, we were able to see rarely and only cytoplasmic EXO70B1 signal in guard cells. This could be due to a lower level of expression of this construct, but could also point to a distinct form of regulation of EXO70B1 protein levels in guard cells.

## Discussion

Mechanisms related to RIN4 function in plant immunity interactions are not fully understood despite quite large efforts from the research community. In this article, we present new data on EXO70B1 localization with respect to its immunity-related function. We show by both Y2H and co-IP assays that the EXO70B1 exocyst subunit interacts with RIN4 and that this interaction is almost lost when RIN4 is cleaved into fragments. This suggests that the interaction site for EXO70B1 lies within the AvrRpt2 protease cleavage motifs of RIN4, or that they at least contribute to the 3D structure recognized by EXO70B1. Furthermore, as the N-terminal RIN4 fragment released to the cytoplasm upon cleavage interacts somewhat more strongly with EXO70B1 than the C-terminal fragment, we might speculate that the relocalization of the N-terminal part of RIN4 may prevent proper PM targeting of EXO70B1 in a dominant negative manner. Indeed, we observed RFP–EXO70B1 signal mainly in the cytoplasm when we co-infiltrated the *P. syringae* strain containing AvrRpt2 protease as compared with the mutant strain control. Although less specific, DTT results further corroborate the hypothesis that EXO70B1 PM localization is dependent, at least in the *N. benthamiana* heterologous system, on the RIN4 protein. This is also in agreement with public proteomic data, according to which EXO70B1 as well as EXO70E2 exocyst subunits are predicted to interact with RIN4 protein (Da Cunha, 2009; Afzal *et al.*, 2013).

Interestingly and in contrast to EXO70B1, its closest homolog EXO70B2 is not recruited to the PM by RIN4. This corresponds well with Y2H data, which showed that EXO70B2 interacts only weakly with the full-length RIN4 protein. Despite the fact the interaction data and quantification of the PM to cytoplasm ratio for EXO70B2 do not support the RIN4-dependent localization hypothesis, we cannot completely rule out a contribution of RIN4 to EXO70B2 localization and function in an immunity context, especially in a homologous Arabidopsis system. EXO70B2 is known to play a role in defense against microbial pathogens (Pecenková *et al.*, 2011; Stegmann *et al.*, 2012). Alternatively, EXO70B2 might use different proteins from the NOI family as adaptors for PM targeting. This would be consistent with the apparent lack of phospatidylinositol-4,5-bisphosphate-binding sites on the C-terminus of EXO70B2 (Zárský *et al.*, 2009), which are responsible for binding to the PM.

Localization of fluorescent constructs showed that only RIN4, but not the aquaporin PIP2;1, targets EXO70B1 to the PM, indicating that RIN4 specifically pulls EXO70B1 to the PM. As the Arabidopsis genome encodes several NOI proteins, these might also contribute to EXO70B1 localization and function; however, there are no NOI proteins predicted that share substantial amino acid similarity with RIN4 in the *N. benthamiana* genome. We were therefore able to show that RIN4 is specifically sufficient to localize EXO70B1 to the PM. Our preliminary data also indicate that NOI6 interacts with EXO70B1 (Supplementary Fig. S1); the precise roles of this and possibly other NOI proteins in exocyst localization have yet to be determined. A similar influence on exocyst localization has been hypothesized for several proteins in different developmental and/or environmental contexts (see below).

Unlike EXO70B1 and EXO70B2 exocyst subunits, SEC6 does not interact with RIN4 in our Y2H assay. Consistently, we did not observe any recruitment of SEC6–RFP to the PM by GFP–RIN4. SEC6 was shown to interact with EXO70B1 (Kulich *et al.*, 2013); however, this Y2H interaction was rather weak and this is probably why we did not observe any SEC6 recruitment even when HA-EXO70B1 was co-infiltrated along with GFP–RIN4. Alternatively, the RFP fluorescent tag on the C-terminus of SEC6 may prevent *in vivo* interaction between HA-EXO70B1 and SEC6–RFP, or SEC6 and RIN4 may compete for a common binding site of EXO70B1. Furthermore, SEC6 may require other Arabidopsis exocyst subunits not present in *N. benthamiana* for its interaction and subsequent recruitment. Unlike *N. benthamiana*, in Arabidopsis SEC6–RFP localizes to the PM and its localization is EXO70A1 dependent (Fendrych *et al.*, 2013). Therefore, we cannot currently assess the contribution of RIN4 to exocyst holocomplex PM recruitment. However, as in the case of ROP2–RIC7 EXO70B1 PM recruitment, the rest of the complex need not follow EXO70B1. The exocyst has been recently described to exist as a stable octameric complex in yeast cells (Heider *et al.*, 2016). Yet, the EXO70 subunit alone is capable of inducing membrane curvature independently of the rest of the complex (Zhao *et al.*, 2013). The notion that particular EXO70 isoforms present in plant cells could act separately to support specific membrane trafficking events (Zárský *et al.*, 2013) would further explain why RIN4 recruits only EXO70B1 but not SEC6.

ROP GTPases are one of the main regulators of cell polarity establishment. The role of GEF and GAP proteins as ROP GTPases regulators in defense against plant pathogens is well established (Huesmann *et al.*, 2011; Yamaguchi and Kawasaki, 2012). The SEC3 subunit interacts with activated ROP GTPase through the ICR1 scaffold protein (Lavy *et al.*, 2007). A recent study also revealed another ROP GTPase-associated protein as an exocyst interactor. RIC7 interacts with active ROP2 GTPase and pulls EXO70B1 to the PM in *Vicia faba* guard cells. Here, RIC7 seems to regulate stomatal opening negatively, probably through negative regulation of EXO70B1 localization and thus function, which is suggested by the retarded light-induced stomatal opening in the *exo70B1-1* mutant (Hong *et al.*, 2016). During arbuscular mycorrhizal symbiosis, plant Vapyrins might also act as scaffold proteins and thus recruit EXO70I to or maintain it at the tip of periarbuscular membranes (Zhang *et al.*, 2015). There is yet another example of exocyst recruitment that has recently been observed in differentiating xylem vessel cells, where the VETH2–COG complex recruits EXO70A1 to cortical microtubules (Oda *et al.*, 2015). Currently it is not known whether the exocyst interacts with any other adaptor proteins besides ICR1, RIC7, and possibly Vapyrins. NOI proteins may be good candidates for exocyst adaptors.

Interestingly, in the WT background, RFP–EXO70B1 localization changes only in stomatal guard cells when GFP–RIN4 is co-expressed in stably transformed Arabidopsis lines. Liu *et al.* (2009) showed that while the *RIN4* gene is expressed more in stomatal cells than in the whole leaf, the RIN4 protein is less abundant in stomata than in the whole leaf. Therefore, overexpressing *RIN4* under the ubiquitin promoter may enhance the level of RIN4 protein in guard cells and induce the recruitment of EXO70B1 to the PM. The fact that GFP–RIN4 recruits RFP–EXO70B1 to the PM only in guard cells might also suggest a distinct form of regulation. Here, the ROP2–RIC7 pathway was also shown to influence EXO70B1 PM localization (Hong *et al.*, 2016; see above). Our results seem to contradict this report, showing that RFP–EXO70B1 localized to the cytoplasm and nucleus in open stomatal guard cells in Arabidopsis seedlings. In contrast, Hong *et al*. reported a recruitment of DsRed-labeled EXO70B1 to the PM in open stomata in the light in *V. faba*. However, Hong *et al*. did observe EXO70B1 in the cytoplasm of open stomatal guard cells when they used a C-terminal sGFP fusion in Arabidopsis. Also, unlike our study, they used the constitutive 35S promoter for their overexpression studies. Nevertheless, our data suggest an additional layer of regulation of EXO70B1 localization in guard cells. Besides ROP2–RIC7, RIN4 seems to influence EXO70B1 localization in guard cells with possible implications in plant–pathogen interaction. A very recent report has also suggested abscisic acid-dependent EXO70B1 ubiquitination and degradation in guard cells (Seo *et al.*, 2016), which further highlights the complexity of the regulation of EXO70B1 function in stomata.

What might be the biological role for PM recruitment of EXO70B1? Considering the roles of EXO70B1 in plant immunity and autophagic transport (Kulich *et al.*, 2013; Stegmann *et al.*, 2013), one might speculate that these two functions are actually connected. There are two lines of evidence that hint at this conclusion. First, the exocyst is known to operate at the PM where it is believed to mediate tethering of defense-related secretory vesicles to the pathogen attack site, where it could co-operate with RIN4. Cleavage of RIN4 by the AvrRpt2 protease would consequently release both RIN4 fragments and EXO70B1 to the cytoplasm, blocking the tethering of secretory vesicles or compartments. Despite some ambiguity of the published data (compare Stegmann *et al.*, 2013; Zhao *et al.*, 2015), the *exo70B1* mutant has been shown by one study to be more resistant to AvrRpt2-expressing *Pseudomonas* (Zhao *et al.*, 2015). We believe this could be due to elevated levels of salicylic acid in this mutant, which, along with the activation of RPS2 resistance protein, triggers a strong hypersensitive response (Kulich *et al.*, 2013). Secondly, multivesicular bodies, which have been described to fuse with the PM at the pathogen attack site as a means of defense (An *et al.*, 2006), are speculated to originate with a contribution from the autophagy pathway (Katsiarimpa *et al.*, 2013; Kulich *et al.*, 2013). Thus, we speculate that EXO70B1 is recruited to the PM by RIN4, where it can participate in the tethering of secretory vesicles or autophagic compartments to the pathogen attack site. We are currently investigating which compartments are involved in this defense pathway, and to what extent the autophagic function of EXO70B1 contributes to this pathway. Upon flg22 treatment, the cleavage of RIN4 by AvrRpt2 was shown to release RIN4 fragments that suppress callose deposition and support enhanced growth of the *P. syringae hrcC* mutant (Afzal *et al.*, 2011). Currently, we cannot say whether EXO70B1 contributes to this pathogen-induced callose deposition. Further research is obviously needed to gain more insight into how secretory and autophagy pathways in plant cells are co-ordinated to ward off microbial pathogens.

## Supplementary data

Supplementary data are available at *JXB* online.

Fig. S1. Yeast-two hybrid assays showing the interaction between exocyst subunits and NOI6.

Fig. S2. SYP121 does not recruit EXO70B1 to the plasma membrane in *N. benthamiana* leaf cells.

Fig. S3. EXO70B1–mRuby2 localizes to the PM and cytoplasm in both WT and the *rin4 rps2* mutant.

## Supplementary Material

Supplementary_Figures_S1_S3
