# Supplementary material for: RIN4 recruits the exocyst subunit EXO70B1 to the plasma membrane
Source: J Exp Bot. 2017 Feb 20;68(12):3253–65. doi: 10.1093/jxb/erx007 (PMC5853926; doi:10.1093/jxb/erx007)
Supplement: Supplementary_Figures_S1_S3 [file erx007_suppl_supplementary_figures_s1_s3.pdf]

Supplementary Figure S1

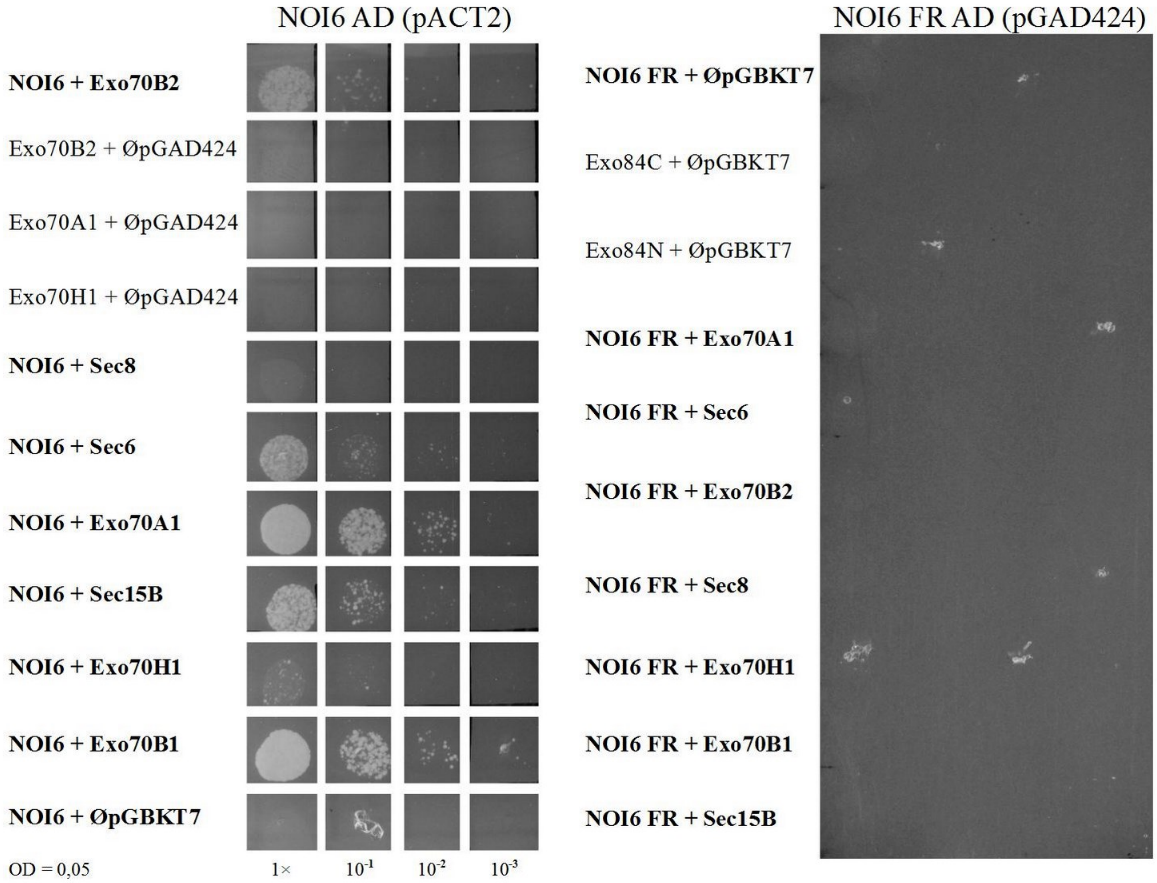

Figure S1. Yeast-two hybrid assays showing interaction of exocyst subunits with NOI6.
Full length NOI6 protein (on the left) and C-terminal membrane anchored NOI6
fragment (on the right).

Supplementary Figure S2

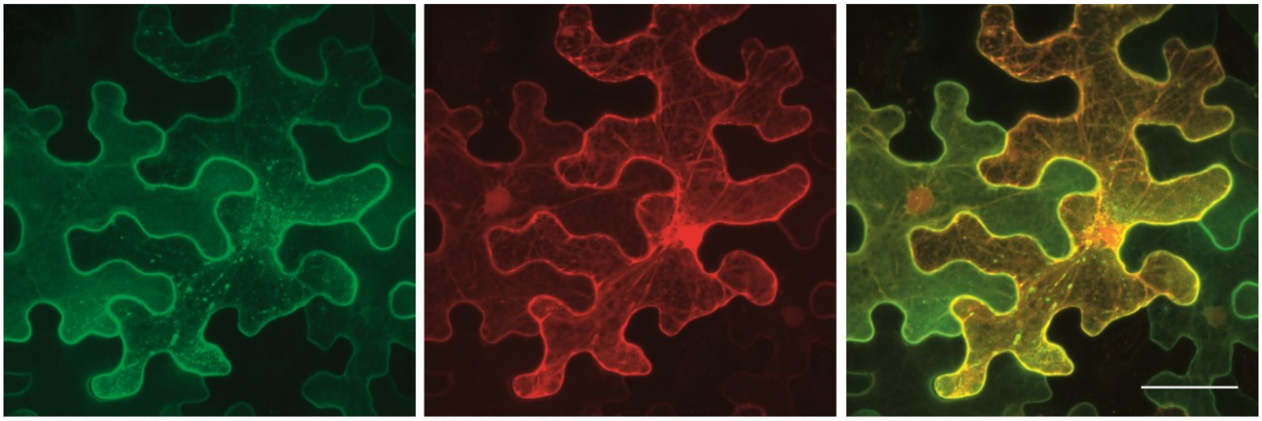

Figure S2. SYP121 does not recruit EXO70B1 to the plasma membrane in N. benthamiana leaf cells. GFP-SYP121 expressed under the ubiquitin promoter (left) was coexpressed with RFP-EXO70B1 under the ubiquitin promoter (middle). Shown is the z projection of 33 confocal images. Merged image is on the right. Scale bar = 50 μm.

Supplementary Figure S3

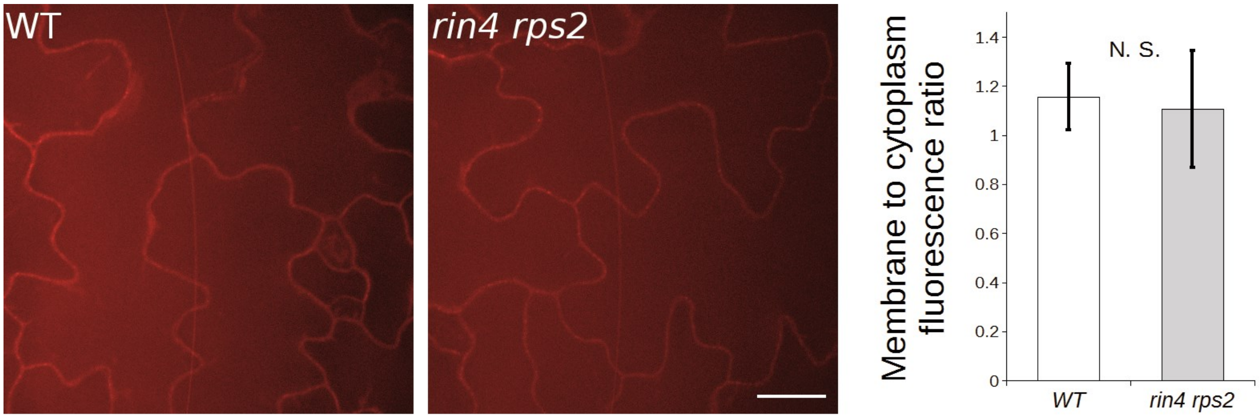

Figure S3. EXO70B1-mRuby2 localizes to the PM and cytoplasm in both WT and *rin4 rps2* mutant. Shown are the confocal images from the spinning disc microscope. EXO70B1-mRuby2 construct was expressed under the native EXO70B1 promoter in Arabidopsis cotyledon cells. Quantification of membrane to cytoplasm fluorescence ratio is shown on the right. Mean and SD (error bars) were calculated from 14 cells. Differences between the means are not statistically significant (N.S.; t-test p value = 0.501). Scale bar = 20 μm.
